# Managers perception of hospital employees’ effort-reward imbalance

**DOI:** 10.1186/s12995-023-00376-4

**Published:** 2023-06-06

**Authors:** Meike Heming, Johannes Siegrist, Rebecca Erschens, Melanie Genrich, Nicole R. Hander, Florian Junne, Janna K. Küllenberg, Andreas Müller, Britta Worringer, Peter Angerer

**Affiliations:** 1 Institute of Occupational, Social, and Environmental Medicine, Centre for Health and Society, Faculty of Medicine, Heinrich-Heine University Düsseldorf, Moorenstraße 5, 40225 Düsseldorf, Germany; 2 Institute of Medical Sociology, Centre for Health and Society, Faculty of Medicine, Heinrich-Heine University Düsseldorf, Moorenstraße 5, 40225 Düsseldorf, Germany; 3 Department of Psychosomatic Medicine and Psychotherapy, University Hospital Tuebingen, University of Tuebingen, 72076 Tuebingen, Baden-Wuerttemberg, Germany; 4 Institute of Psychology, Work and Organisational Psychology, University of Duisburg-Essen, Universitätsstr. 2, 45141 Essen, Germany; 5 Department of Psychosomatic Medicine and Psychotherapy, Ulm University Medical Center, Albert- Einstein-Allee 23, 89081 Ulm, Germany; 6 University Clinic for Psychosomatic Medicine and Psychotherapy, University Medicine, Otto-von-Guericke University Magdeburg, Leipziger Str. 44, 39120 Magdeburg, Germany; 7 Institute for Medical Psychology, Centre for Psychosocial Medicine, University Heidelberg, University Hospital Heidelberg, Bergheimer Straße 20, 69115 Heidelberg, Germany; 8 Institute for Research and Development of Collaborative Processes, FHNW School of Applied Psychology, Riggenbachstrasse 16, Olten, 4600 Switzerland

**Keywords:** Effort-reward imbalance, Criterion validity, External assessment, Hospital, Managers, Employees

## Abstract

**Objective:**

Hospitals are frequently associated with poor working conditions that can lead to work stress and increase the risk for reduced employee well-being. Managers can shape and improve working conditions and thereby, the health of their teams. Thus, as a prerequisite, managers need to be aware of their employees’ stress levels. This study had two objectives: At first, it aimed to test the criterion validity of the Effort-Reward Imbalance (ERI) questionnaire measuring psychosocial workload in hospital employees. Secondly, mean scales of the ERI questionnaire filled in by employees were compared with mean scales of an adapted ERI questionnaire, in which managers assessed working conditions of their employees.

**Methods:**

Managers (n = 141) from three hospitals located in Germany assessed working conditions of their employees with an adapted external, other-oriented questionnaire. Employees (n = 197) of the mentioned hospitals completed the short version of the ERI questionnaire to assess their working conditions. Confirmatory factor analyses (CFA) were applied to test factorial validity, using the ERI scales for the two study groups. Criterion validity was assessed with multiple linear regression analysis of associations between ERI scales and well-being among employees.

**Results:**

The questionnaires demonstrated acceptable psychometric properties in terms of internal consistency of scales, although some indices of model fit resulting from CFA were of borderline significance. Concerning the first objective, effort, reward, and the ratio of effort-reward imbalance were significantly associated with well-being of employees. With regard to the second objective, first tentative findings showed that managers’ ratings of their employees’ effort at work was quite accurate, whereas their reward was overestimated.

**Conclusions:**

With its documented criterion validity the ERI questionnaire can be used as a screening tool of workload among hospital employees. Moreover, in the context of work-related health promotion, managers’ perceptions of their employees’ workload deserve increased attention as first findings point to some discrepancies between their perceptions and those provided by employees.

**Supplementary Information:**

The online version contains supplementary material available at 10.1186/s12995-023-00376-4.

## Introduction

The hospital setting as a workplace is frequently associated with poor working conditions, e.g. exposure to excessive mental and emotional demands and high work pressure [1]. This can lead to “toxic” work stress that increases the risk of reduced well-being, mental and physical illnesses [2, 3].

The effort-reward imbalance (ERI) model offers an established stress-theoretical explanation of the links between poor work conditions and reduced health. The model assumes that the recurrent and prolonged experience of failed reciprocity between high effort (the cost) expended in work and the low reward (the gain) received, activates persistent negative emotions of reward frustration and related reward system circuitry in the brain [4, 5]. This is due to the violation of a basic principle of social exchange, namely the equivalence of “giving” and “receiving” in “costly” transactions. Since the brain’s reward circuit is sensitive to the experience of inequality in social exchange, it can activate, permanently or recurrently, the stress axes, especially the hypothalamic-pituitary-adrenal axis, triggering states of stress overload in several regulatory systems of the body. As a result, the risk of developing a stress-related disorder such as coronary heart disease or depression is increased in professionals exposed to an imbalance between performance and reward at work [4, 5].

Empirically, about one quarter of hospital physicians working in surgical fields experienced work stress in terms of an effort-reward imbalance (ER-ratio), which is more than in the general working population in Germany [6]. Negative changes in the psychosocial work environment, specifically increased ER-ratio, were found to be associated with depressive symptoms in German physicians during their hospital training [7]. As an indication that different working conditions are associated with different levels of effort-reward-imbalance in the hospital, a comparative study found differences in work stress and working conditions (e.g. total working hours) to the benefit of migrated German physicians in Sweden compared to physicians in Germany [8]. Another study on physicians in Germany found that 57% of the study participants experienced poor working conditions in terms of an ER-ratio [9]. The study also found that effort, reward and the ER-ratio were associated with poorer perceived quality of patient care [9].

The same associations between ERI and poor health are found in hospital nurses, as several studies have shown, e.g.: A higher ER-ratio was associated with frequent short sickness absence days [10], with depression [11], and with reduced well-being [12–14].

The risk of affective and stress related disorders is elevated in the human services professions, especially in health care workers with nurses being among those with the highest risk [15]. Physicians and nurses suffer from a high prevalence of burnout [16], which can lead to a deteriorated quality of patient care (i.e. in terms of medical errors, [17, 18]) or poor job satisfaction [19, 20]. Against this background, it is important to focus on employee health [21].

In order to improve health and well-being at the workplace, managers have an important role, especially in terms of managing and shaping working conditions or improving job satisfaction [22–26]. For example, perceived transformational leadership style was positively associated with well-being of certain groups of health-care professionals in a hospital [27]. A systematic review on nurse leadership style and nurse satisfaction showed, that nurses reported higher job satisfaction when they worked with relational leaders than with task-oriented leaders [28]. Physician’s well-being and satisfaction was shown to be impacted by the leadership qualities of their immediate supervisors [29]. A higher leadership quality, assessed by the physicians, was associated with a decrease in burnout and increase in satisfaction [29, 30]. Congruence between leadership interest and interests of physicians was associated with a lower level of perceived stress by physicians [31].

It is therefore important that managers are aware of potentially harmful working conditions. We assume that awareness, perception and understanding of poor working conditions is a first step for managers’ motivation to improve their work environment, specifically by implementing health-related interventions [32]. To then successfully implement interventions, managers need to intervene as active participants, informing and encouraging their employees, integrating them in participation and decision-making [33].

However, an early study on leadership behavior showed that leaders’ perceptions of stressors did not always match employee-reported stressors [34]. Yet, a recent qualitative study explored how hospital managers perceived working conditions and mental stress of their employees [35]. In this study, hospital managers reported work stressors pointing to stressful work tasks, such as high emotional demands or lack of decision latitude [35]. Staff shortage was also mentioned as one of the main stressors, and managers’ social support was often reported as an important resource to buffer against stress [35]. The work characteristics mentioned in this study were compatible with relevant dimensions in well-known work stress theories, such as the Job-Demand-Control Support model [36] or the ERI model [4, 35].

With this study, we set out, first, to test criterion validity of the ERI questionnaire among the sample of hospital employees by analysing associations with a standardised measure of subjective well-being [37]. As a second aim, we demonstrate comparisons of mean scale scores of the original ERI scales (among employees) with scores of the adapted version of managers’ ERI scales, reflecting their perceptions of employees’ workload.

## Methods

### Study design and participants

This cross-sectional study used baseline data from a cluster-randomized trial called „Mental Health in the workplace hospital (Acronym “SEEGEN” in German). SEEGEN aimed to evaluate the effects of five behavioral and organizational interventions on mental health and well-being of hospital employees in three different hospitals located in Germany [38]. Detailed information on the study design has been published [38].

Potential participants were all employees of the three hospitals, working in either managerial or employee positions, in the 18 cluster units (6 clusters per hospital) and being involved in patient care. Potential occupational groups were medical services (i.e. physicians), nursing services (i.e. nurses) or functional service and other (i.e. physiotherapists, diagnostics or secretariats involved with patient care). Of the three participating hospitals, one was a university hospital, one was a community hospital, and a private health company owned the third one. Interested employees received verbal and written information about the study process, the interventions, and the evaluation. Participants gave written informed consent and could participate voluntarily and terminate their participation in the study at any time [38]. The ethics committees of respective universities involved approved the study (Ulm University April 2019 (Application No. 501/18), Heidelberg University September 2019 (Application No. S-602/2019) and Heinrich-Heine University Düsseldorf September 2019 (Application No. 613R). The study was registered in the German Clinical Trials Register (DRKS00017249).

The baseline recruitment took place from October 2019 until March 2020. Inclusion criteria were willingness to participate in the study, to complete questionnaires at three timepoints, being aged betweeen 18 and 70 years, sufficient German language skills and employment in patient care in the participating hospitals. Questionnaires asked about sociodemographic factors, organizational indicators such as job satisfaction, or intention to leave, and questions about mental health and well-being. The questionnaires could be filled in either paper-based or electronically and were stored on a server at the Institute of Medical Biometry and Informatics (IMBI) in Heidelberg, Germany [38].

Within the cluster-randomized trial, N = 5654 individuals were eligible to take part. Out of these, 462 participants gave informed consent to participate in the study. At baseline (T0), 407 individuals filled in questionnaires (response rate 88.1%). Some questions were completed by all employees, while other sections were only for employees with leadership responsibility. In the present study, participants are divided into two different analytical samples representing either employees or managers. Participants are considered as managers if they had a top or middle management position. A top management position refers to having ultimate responsibility for the management of an entire unit, such as chief physician. A middle management position refers to leadership responsibility for subunits, such as senior physician, nursing manager or team leader. In case of missing data on the hierarchy level, participants who reported in the inclusion form of the baseline questionnaire that they had a leading position were also categorized as managers (n = 162). The remaining participants were considered employees (n = 245). The final study samples consist of participants with valid data for all relevant study variables without any missing data. This results in n = 141 managers and n = 197 employees.

### Measures

#### Development of adapted effort-reward-imbalance (ERI) scale – short version for managers

It was aimed to adapt the questionnaire only with very few changes so that it remains close to the original, psychometrically tested short ERI version [39]. Therefore, content and rating procedure of all scale items were maintained in the adapted version. Nevertheless, some language adaptation was required. To this end, a team of four authors of this manuscript (PA, AM, BW, MG) discussed and agreed on subtle language changes in an effort to accurately reflect the employees’ perspective, as assessed by managers. The final version was approved by the author of the original ERI questionnaire (JS). As a next step, the cluster-randomized trial started with a pilot-workshop (prior to the baseline questionnaire), sensitizing managers of the participating hospitals regarding stressors and resources at the workplace. Within this workshop, participants were asked about comprehensiveness of items. As no problems of interpretation evolved, this version was subsequently applied in the baseline wave (for an English version of the questionnaire see Additional File 1).

#### Effort-reward-imbalance (ERI) scale – short version for self-assessment of employees

Employees filled in the short version of the standard ERI questionnaire [39]. Here, similarly, effort was measured with three items and reward was measured with seven items. Answers were given on a 4-point Likert scale, where one indicated strong disagreement and four strong agreement. Higher values of scores of the two scales reflected higher effort and higher reward, respectively. In addition, a ratio of the two scales was calculated to quantify imbalance between effort and reward at individual level, adapted for unequal number of items. Here, a value of 1.0 indicated a balance between ‘cost’ and ‘gain’. Accordingly, values beyond 1.0 were defined as exposure to stressful workload.

#### Subjective well-being

Employees filled in the World Health Organization Well-Being Index (WHO-5) [37]. Five items are answered on a 6-point Likert scale, where zero indicates “at no time” and five indicates “all of the time”. To achieve a total score, the items are summed up and multiplied by four. Thus, the scale ranges from zero to 100. Higher values indicate a better well-being.

#### Statistical analyses

To test the psychometric properties of the adapted ERI questionnaire for managers and of the standard ERI questionnaire for employees, item and scale means were calculated. To assess internal consistency Cronbach’s alpha and item-total correlations were computed. Beforehand, it was decided to eliminate the item measuring job insecurity in both questionnaires as this aspect is not relevant in the hospital setting of this country (high job security). A further item measuring the respect received from the superior was excluded from final analyses due to insufficient item characteristics and problematic assessment by managers. The corrected item-total correlation for both items was less than 0.30 assessed by managers and the item measuring respect received from the superior had a corrected item-total correlation below 0.30 in employees, which also supported removing the items. In a next step, we conducted confirmatory factor analyses to test the factorial structure of the theoretical constructs. The following goodness-of-fit indices were used: The goodness-of-fit index (GFI, acceptable fit for values > 0.90), the adjusted-goodness-of-fit index (AGFI, acceptable fit for values > 0.85), the Root Mean Square Error of Approximation (RMSEA, adequate fit for values between 0.05 and 0.08) and the comparative fit index (CFI, values > 0.95 indicate an acceptable fit) [40]. A two-factor-model was estimated where the respective items loaded on the first-order factors ‘effort’ and ‘reward’.

We performed analyses in order to investigate criterion validity of the standard ERI instrument among hospital employees. Therefore, we calculated multiple linear regression analyses in which subjective well-being (WHO-5) was set as the outcome and the respective ERI scales were set as the independent variables. The analyses were adjusted for age, sex, marital status, employment status and occupational position. Results are presented as unstandardized regression coefficients with 95% confidence intervals (CIs). A p-level below 0.05 was considered as the significance level.

In addition, we show mean scale scores of the original ERI scales (among employees) and scores of the adapted ERI scales assessed by managers, reflecting the perception of employees’ workload. These comparisons are given at the level of the three participating hospitals, and additionally at the level of clusters (Additional file 2). R Statistical Software v. 4.1.3 was used to conduct confirmatory factor analysis and all other analyses were performed with SPSS v. 27.

## Results

### Descriptive statistics

Table 1 shows descriptive statistics for the two study samples of employees (n = 197) and managers (n = 141). Around 83% of the employees were female, while 60% of the managers were female. The age distribution in the employee sample was relatively balanced, while in the sample of managers 46% were aged 51 or older. Most participants were married or in partnership (71.7% and 75.9%). Almost half of the employees were working parttime. Of the managers, 19% were working parttime. While 58% of the employees were working in nursing service, 36% of the managers were employed in nursing service.

**Table 1:** Descriptive statistics for study variables of the two study samples

	Employees (n = 197)	Managers (n = 141)
	n (%)	n (%)
**Age**		
21–30	51 (25.9)	10 (7.1)
31–40	50 (25.4)	30 (21.3)
41–50	40 (20.3)	36 (25.5)
51+	56 (28.4)	65 (46.1)
**Sex**		
female	163 (82.7)	85 (60.3)
Non-female	34 (17.3)	56 (39.7)
**Marital status**		
Married/in partnership	140 (71.7)	107 (75.9)
Single/divorced	57 (28.9)	34 (24.1)
**Employment status**		
Full time	102 (51.8)	114 (80.9)
Part time	95 (48.2)	27 (19.1)
**Occupational position**		
Medical service	39 (19.8)	58 (41.1)
Nursing service	115 (58.4)	50 (35.5)
Functional service + other	43 (21.8)	33 (23.4)

### Factor validity

Confirmatory factor analyses were conducted to test factorial validity of the ERI questionnaires for employees and managers. Table 2 shows the factor pattern and model fit of these confirmatory factor analyses. Overall, the models did not reach an acceptable level of fit.

Despite these findings, item-total correlations and Cronbach’s alpha were calculated for both questionnaires (data not shown). Cronbach’s alpha for effort was 0.83 and for reward 0.73. Item-total correlations varied from 0.35 to 0.76 for the ERI questionnaire assessed by managers. For employees, Cronbach’s alpha for effort was 0.7 and for reward 0.69. Item-total correlations varied from 0.39 to 0.61. An additional conducted confirmatory factor analysis for the ERI questionnaire assessed by managers resulted in far better model fit indices, when item ERI5 (“The job promotion prospects for my employees are poor.”) was removed from the analysis. Within these analyses GFI increased to 0.937, AFGI to 0.864 and CFI to 0.930 while RMSEA decreased to 0.113 (CI 0.071–0.158, data not shown).

**Table 2:** Factor loadings and goodness-of-fit of confirmatory factor analyses of ERI questionnaires

	Employees (n = 197)	Managers (n = 141)
	Standardized factor loadings	Standardized factor loadings
**Effort**		
Eri1	0.798	0.944
Eri2	0.637	0.707
Eri3	0.583	0.707
**Reward**		
Eri5	0.454	0.364
Eri6	0.507	0.589
Eri8	0.604	0.709
Eri9	0.714	0.661
Eri10	0.552	0.654
**GFI** ^**a**^	0.919	0.893
**AFGI** ^**b**^	0.847	0.797
**RMSEA** ^**c**^ **(90% CI)**	0.119 (0.090–0.149)	0.145 (0.112–0.180)
**CFI** ^**d**^	0.839	0.853

^a^ Goodness-of-fit index.

^b^ Adjusted goodness-of-fit index.

^c^ Root Mean Square Error of Approximation.

^d^ Comparative fit index.

Presented are values for the two different questionnaires for employees and managers.

### Criterion validity

Results of multiple linear regression analyses estimating the association between ERI scales and subjective well-being of employees are shown in Table 3. High efforts were significantly negatively associated with well-being (B=-2.337, p = 0.005, Model 2, Table 3). Thus, employees who reported to perceive higher efforts had lower well-being compared to employees who reported to perceive lower efforts. Rewards were significantly positively associated with well-being of employees. Employees, who reported to receive higher rewards at work, reported higher well-being compared to employees who reported to receive lower rewards. The ER-Ratio showed a significant negative assocation with well-being of employees. Employees, who had a higher ER-Ratio (i.e. a greater imbalance of effort and reward), had lower well-being compared to employees who had a lower ER-Ratio.

**Table 3:** Results from separate multiple linear regression analyses estimating the association between ERI and WHO-5 of employees (n = 197)

	WHO-5								
	Crude Model^a^			Model 1^b^			Model 2^c^		
	B^e^	p-value	95% CI	B^e^	p-value	95% CI	B^e^	p-value	95% CI
**Effort**	**-3.224**	< 0.001	-4.741;-1.707	**-3.197**	< 0.001	-4.754;-1.639	**-2.337**	0.005	-3.958;-0.716
**Reward**	**2.054**	< 0.001	1.090;3.018	**2.187**	< 0.001	1.164;3.210	**1.662**	0.002	0.593;2.730
**ER-Ratio** ^**f**^	**-9.382**	< 0.001	-13.317:-5.448	**-9.646**	< 0.001	-13.764;-5.528			

^a^ unadjusted model.

^b^ adjusted for age and sex, marital status, employment status, occupational position.

^c^ adjusted for age, sex, marital status, employment status, occupational position and either effort or reward.

^e^ unstandarized regression coefficient.

^f^ Effort-Reward-Imbalance Ratio.

Bold values indicate a p-value below 0.05.

### Comparison of ERI mean scales

Table 4 shows a comparison of ERI mean scales for the two different questionnaires by the three participating hospitals. As can be seen, managers’ perceptions of employees’ effort match employees’ perceptions quite well, whereas their ratings of employees’ rewards are consistently higher. In consequence, the mean ER-Ratio, quantifying the stressful experience of an imbalance between cost and gain at work, is lower among managers’ assessment.

**Table 4:** Descriptive ERI means of employees and managers in the participating hospitals

	Hospital 1				Hospital 2				Hospital 3			
	Employees (n = 51)		Managers (n = 32)		Employees (n = 91)		Managers (n = 48)		Employees (n = 55)		Managers (n = 61)	
ERI^a^	Mean	SD^c^	Mean	SD^c^	Mean	SD^c^	Mean	SD^c^	Mean	SD^c^	Mean	SD^c^
Effort	10.33	1.58	10.28	1.71	10.05	1.84	10.42	1.57	10.02	1.64	9.93	1.76
Reward	11.08	2.39	12.88	2.24	10.51	2.53	12	2.54	11.18	3.20	11.97	2.74
ER-Ratio^b^	1.68	0.68	1.39	0.43	1.72	0.64	1.54	0.52	1.66	0.66	1.49	0.55

^a^ Effort-Reward Imbalance Questionnaires. Presented are values for the two different questionnaires for employees and for managers.

^b^ Effort-Reward-Imbalance Ratio.

^c^ Standard Deviation.

Additional findings of ERI mean scales for the two different questionnaires by the cluster units can be found in the additional material (Additional file 2). Mean values of ER-Ratios, assessed by managers about their employees, were lower than mean values of ER-Ratios assessed by employees’ self-rating in 15 out of 18 participating clusters.

## Discussion

This study aimed to investigate criterion validity for the ERI model within a hospital setting and to compare an external assessment of working conditions of hospital employees by their managers with hospital employees’ self-reported responses.

Results of internal consistency showed acceptable results for both questionnaires. For example, the corrected item-total correlations and internal consistency were acceptable for both ERI questionnaires. Cronbach’s alpha values of the ERI questionnaire were somewhat lower compared to earlier studies [39, 41, 42], but still comparable to other studies in the healthcare setting [12, 43]. However, confirmatory factor analyses showed some model fit indices that were of borderline significance. Our study sample was relatively small in comparison to other studies performing confirmatory factor analyses, which could in part explain a restricted model fit.

Interestingly, despite a relatively crude assessment method in case of managers, we found some discrepancies in the perception of employees’ workload by managers and the self-reported workload of employees. While managers perceived their employees’ efforts rather accurately, as indicated by similar mean scores of the scale, they perceived higher rewards than those reported by employees themselves. Accordingly, managers’ assessment of their employees’ overall level of stressful work (the ER-ratio) was lower than the one resulting from employees’ self-reports. Given the preliminary assessment method applied to managers, these findings need to be discussed very carefully. While it is rather unlikely that employees failed to assess their rewards in an accurate way, it seems probable that managers overestimate the rewarding working conditions of their employees. For instance, they may have internalized a perspective of dominance and control, legitimizing established employment terms, including occupational rewards. This perceptual adaptation reduces their empathy towards experienced inequity among subordinates. In line with this interpretation, a previous study reported that managers who overestimated their supportive behaviour were less sensitive to the concerns of their employees [44]. Overestimating rewards, including the non-material rewards provided by the managers’ own leadership behaviour, matches a strong tendency towards positive self-evaluation [45].

Importantly, if managers in hospitals are not aware of their employees’ need for reward and cannot adequately assess their own behavior (i.e. giving out rewards to employees), they might not perceive the necessity to improve working conditions.

### Strengths and limitations

Several limitations need to be considered. Due to the small sample size, an acceptable model fit of the data was difficult to be met. Moreover, a high heterogeneity of professional groups within the sample may have weakened the scale’s internal consistency. A major limitation concerns the other-directed assessment of employees’ workload by managers. The crude method does not indicate on what empirical basis these judgments were made, whether specific individuals served as reference persons, or whether evaluations were influenced by social desirability. Despite our efforts to compare the two groups at the level of single hospital departments (“cluster”), a precise matching was not possible. Clearly, given the methodological challenges of assessing the workload of other people, a more sophisticated measurement approach is expected from future research on this topic. Concerning the findings on criterion validity, common method variance in cross-sectional studies with self-rated data measuring independent and dependent variables define a further methodological challenge [46]. Finally, the study design yields the possibility that either very stressed or non-stressed participants were overrepresented. In the first case, they may have been motivated to participate in a stress-reducing activity, or in the second case, they may have agreed to participate due to more free time as they were more relaxed from work. These limitations were balanced by a particular strength of this study as this is one of the rare reports contrasting self-reports of subordinate groups of employees with external assessments of employees’ efforts and rewards by their managers. Moreover, in either case, workload was assessed by a theoretically grounded, psychometrically well-tested method, the ERI questionnaire, whose criterion validity was further supported.

### Implications

Although it is widely acknowledged that leadership skills are crucial in healthcare, managers are often ill-prepared for their roles as managers and are lacking appropriate training [47]. In a qualitative study, a majority of residents and supervisors reported a high need for leadership training [48]. A review has shown that among nurses, overall relational-focused leadership styles were associated with improved well-being and a better working environment, compared to task-focused leadership styles [49]. Within a relation-oriented leadership, leaders encourage and support their employees with maintaining relationships, showing appreciation and support [48]. Leadership trainings should therefore prioritize teaching relational competences and also combining organizational and behavioral approaches [50]. In light of this, we believe that managers who have self-awareness and relational or emotional skills are better prepared to assess their employees’ concerns. Thus, it seems important to provide trainings or management courses for managers in hospital settings that focus on these relation-oriented leadership styles [49] and enhance an awareness development process by regarding team leaders’ reflections on their own position and leadership role [51].

## Conclusions

This study showed that higher perceived imbalances of effort and reward were associated with lower self-reported well-being in hospital employees. In addition, some difference between external, other-oriented and self-reported assessment of stressful work, comparing data from managers and subordinated employees, based on the model of effort-reward imbalance at work, were observed. Our results support further investigations into the effects of improved relation-oriented leadership style on employees’ well-being in hospital settings.

## Electronic supplementary material

Below is the link to the electronic supplementary material.

Supplementary Material 1

Supplementary Material 2

## Data Availability

The datasets generated for this study are not publicly available due to data protection guidelines.
