# Supplementary material for: Managers perception of hospital employees’ effort-reward imbalance
Source: J Occup Med Toxicol. 2023 Jun 6;18:8. doi: 10.1186/s12995-023-00376-4 (PMC10246112; doi:10.1186/s12995-023-00376-4)
Supplement: Supplementary file 1 — Supplementary Material 1 [file 12995_2023_376_MOESM1_ESM.docx]

**Additional file 1**

Additional file 1. Self-translated version of the adapted Effort-Reward Imbalance (ERI) Scale – short version for managers.

| 1. | My employees have constant time pressure due to a heavy work load. |
| --- | --- |
| 2. | My employees have many interruptions and disturbances while performing their job. |
| 3. | Over the past few years, the job has become more and more demanding for my employees. |
| 4. | My employees receive the respect they deserve from their supervisors or a respective relevant person. |
| 5. | The job promotion prospects for my employees are poor. (RC) |
| 6. | I have experienced or I expect to experience an undesirable change in the work situation for my employees. (RC) |
| 7. | My employees‘ job security is poor. (RC) |
| 8. | Considering all the efforts and achievements made by my employees, I think they receive the respect and prestige they deserve at work. |
| 9. | Considering all the efforts and achievements made by my employees, I think their job promotion prospects are adequate. |
| 10. | Considering all the efforts and achievements made by my employees, I think their salary/income is adequate. |

RC: Reverse coding.
