# Supplementary material for: Managers perception of hospital employees’ effort-reward imbalance
Source: J Occup Med Toxicol. 2023 Jun 6;18:8. doi: 10.1186/s12995-023-00376-4 (PMC10246112; doi:10.1186/s12995-023-00376-4)
Supplement: Supplementary file 2 — Supplementary Material 2 [file 12995_2023_376_MOESM2_ESM.pdf]

Additional file 2. Descriptive ERI means of employees and managers in the participating cluster units.

**Cluster 1**		**Employees (n=14)**		**Managers (n=9)**	
		**Mean**	**SD[c]**	**Mean**	**SD[c]**
	**ERI[a]**				
	Effort	10.93	1.82	10.44	1.51
	Reward	10.57	2.38	11.44	1.94
	ER-Ratio[b]	1.84	0.71	1.56	0.34
**Cluster 2**		**Employees (n=6)**		**Managers (n=7)**	
		**Mean**	**SD[c]**	**Mean**	**SD[c]**
	**ERI[a]**				
	Effort	9.00	2.19	8.14	2.04
	Reward	13.50	3.94	14.57	2.94
	ER-Ratio[b]	1.28	0.71	1.00	0.41
**Cluster 3**		**Employees (n=7)**		**Managers (n=10)**	
		**Mean**	**SD[c]**	**Mean**	**SD[c]**
	**ERI[a]**				
	Effort	9.43	1.72	10.20	1.75
	Reward	12.43	2.64	12.70	1.70
	ER-Ratio[b]	1.37	0.60	1.37	0.31
**Cluster 4**		**Employees (n=21)**		**Managers (n=5)**	
		**Mean**	**SD[c]**	**Mean**	**SD[c]**
	**ERI[a]**				
	Effort	10.52	1.57	9.40	1.52
	Reward	11.38	2.65	14.60	1.82
	ER-Ratio[b]	1.70	0.76	1.09	0.24
**Cluster 5**		**Employees (n=2)**		**Managers (n=2)**	
		**Mean**	**SD[c]**	**Mean**	**SD[c]**
	**ERI[a]**				
	Effort	9.50	2.12	10.50	2.12
	Reward	12.00	1.41	12.00	0.00
	ER-Ratio[b]	1.31	0.14	1.46	0.29
**Cluster 6**		**Employees (n=4)**		**Managers (n=4)**	
		**Mean**	**SD[c]**	**Mean**	**SD[c]**
	**ERI[a]**				
	Effort	10.00	1.41	10.25	2.87
	Reward	11.50	1.91	12.50	1.00
	ER-Ratio[b]	1.50	0.42	1.39	0.46
**Cluster 7**		**Employees (n=12)**		**Managers (n=9)**	
		**Mean**	**SD[c]**	**Mean**	**SD[c]**
	**ERI[a]**				
	Effort	10.58	1.31	10.11	1.62
	Reward	10.58	2.31	11.44	2.60
	ER-Ratio[b]	1.78	0.62	1.60	0.71
**Cluster 8**		**Employees (n=10)**		**Managers (n=9)**	
		**Mean**	**SD[c]**	**Mean**	**SD[c]**
	**ERI[a]**				
	Effort	10.10	1.37	10.89	1.54

	Reward	10.70	2.63	12.11	3.55
	ER-Ratio[b]	1.70	0.64	1.61	0.52
**Cluster 9**		**Employees (n=8)**		**Managers (n=9)**	
		**Mean**	**SD[c]**	**Mean**	**SD[c]**
	**ERI[a]**				
	Effort	10.63	2.20	9.89	1.62
	Reward	9.13	2.90	12.78	1.72
	ER-Ratio[b]	2.20	0.99	1.31	0.28
**Cluster 10**		**Employees (n=19)**		**Managers (n=6)**	
		**Mean**	**SD[c]**	**Mean**	**SD[c]**
	**ERI[a]**				
	Effort	10.53	1.71	10.67	1.37
	Reward	9.63	1.89	11.00	2.53
	ER-Ratio[b]	1.91	0.55	1.71	0.55
**Cluster 11**		**Employees (n=13)**		**Managers (n=3)**	
		**Mean**	**SD[c]**	**Mean**	**SD[c]**
	**ERI[a]**				
	Effort	9.85	2.12	12.00	0.00
	Reward	8.69	2.78	9.00	2.65
	ER-Ratio[b]	2.05	0.81	2.38	0.83
**Cluster 12**		**Employees (n=5)**		**Managers (n=9)**	
		**Mean**	**SD[c]**	**Mean**	**SD[c]**
	**ERI[a]**				
	Effort	9.60	1.82	10.89	1.36
	Reward	10.20	1.30	13.00	3.04
	ER-Ratio[b]	1.60	0.43	1.50	0.54
**Cluster 13**		**Employees (n=10)**		**Managers (n=14)**	
		**Mean**	**SD[c]**	**Mean**	**SD[c]**
	**ERI[a]**				
	Effort	9.70	2.06	10.00	1.80
	Reward	11.40	3.37	12.14	2.03
	ER-Ratio[b]	1.62	0.81	1.42	0.40
**Cluster 14**		**Employees (n=5)**		**Managers (n=6)**	
		**Mean**	**SD[c]**	**Mean**	**SD[c]**
	**ERI[a]**				
	Effort	9.40	1.82	9.00	0.00
	Reward	12.20	3.70	11.83	2.48
	ER-Ratio[b]	1.43	0.62	1.31	0.26
**Cluster 15**		**Employees (n=18)**		**Managers (n=12)**	
		**Mean**	**SD[c]**	**Mean**	**SD[c]**
	**ERI[a]**				
	Effort	9.39	1.65	10.25	1.71
	Reward	11.67	2.11	13.17	2.12
	ER-Ratio[b]	1.38	0.36	1.34	0.39
**Cluster 16**		**Employees (n=11)**		**Managers (n=3)**	
		**Mean**	**SD[c]**	**Mean**	**SD[c]**
	**ERI[a]**				
	Effort	10.36	1.12	11.00	1.73

	Reward	12.00	1.34	11.00	2.65
	ER-Ratio[b]	1.46	0.22	1.76	0.65
**Cluster 17**		**Employees (n=12)**		**Managers (n=16)**	
		**Mean**	**SD[c]**	**Mean**	**SD[c]**
	**ERI[a]**				
	Effort	10.42	1.31	10.38	1.63
	Reward	10.42	3.63	10.94	2.49
	ER-Ratio[b]	1.84	0.60	1.70	0.61
**Cluster 18**		**Employees (n=20)**		**Managers (n=8)**	
		**Mean**	**SD[c]**	**Mean**	**SD[c]**
	**ERI[a]**				
	Effort	9.95	1.85	10.13	1.64
	Reward	10.75	2.49	11.88	3.68
	ER-Ratio[b]	1.66	0.62	1.59	0.70

[a] Effort-Reward Imbalance Questionnaires. Presented are values for the two different questionnaires for employees and for managers.

[b] Effort-Reward-Imbalance Ratio.

[c] Standard Deviation.
